# Idiopathic CRMO and *MEFV* Gene Variant Alleles: Is There Any Relationship?

**DOI:** 10.1155/2019/9847867

**Published:** 2019-03-27

**Authors:** Farhad Salehzadeh, Hassan Anari, Sepehr Sarkhanloo

**Affiliations:** ^1^Professor in Pediatric Rheumatology, Pediatric Department, Bouali Children's Hospital, Ardabil University of Medical Sciences (ARUMS), Ardabil, Iran; ^2^Assistant Professor in Radiology, Imam Khomeini Hospital, Ardabil University of Medical Sciences (ARUMS), Ardabil, Iran; ^3^Pediatric Department, Bouali Children's Hospital, Ardabil University of Medical Sciences (ARUMS), Ardabil, Iran

## Abstract

**Background and Objective:**

CRMO is an inflammatory disease of bone that occurs more often in children. The clinical manifestations are intermittent fever, pain, and bone lesions, especially in long bones. Although there is an idiopathic type of disease, it is usually associated with some autoimmune disorders. This study evaluates *MEFV* gene mutations as background pathology of idiopathic CRMO.

**Methods:**

Blood samples of patients, who diagnosed as childhood idiopathic CRMO by imaging and pathologic study from June 2011 until September 2018, have been screened for the 12 common pathogenic variants of *MEFV* gene mutations.

**Result:**

Nine patients enrolled in this study, and eight of them were male. The most common involvement locations were tibia and femur, and the least ones were zygoma, calcaneus, and radius. The mean duration of the involvement was 1.3 years. Six patients had only 1 involved location, 2 patients showed two sites of involvement, and one patient had three affected areas. There were two positive *MEFV* gene mutations (22%), as E148Q/wt and K695R/wt both in the heterozygote form. There was no meaningful relationship between *MEFV* gene mutations and the age of onset, gender, and location of involvement. Patients with positive mutation had more involved sites and long duration of involvement significantly.

**Conclusion:**

There is no significant immunopathogenic relationship between the common *MEFV* gene variant alleles and CRMO disease.

## 1. Introduction

CRMO (chronic recurrent multifocal osteomyelitis) also known as chronic nonbacterial osteomyelitis is an inflammatory disease of the bone that frequently occurs in children [1, 2]. CRMO was first defined by Giedion et al. in 1972 [3]. In the past, CRMO was only known as a child-specific disease but nowadays has been diagnosed in adults as well [2]. The condition is more likely to be between the ages of 4 and 14 years (on average, ten years). The frequency of CRMO is 1 : 1,000,000, and the ratio of female to male is 5 to 1 [3]. Clinical manifestations are intermittent fever, pain, and bone lesions that can occur in all bones of the body, especially long bones.

CRMO is known by chronic bone pain and often diagnosed following a lengthy process by bone biopsy and MRI.

This condition is characterized by episodes of systemic inflammation, including serological inflammation markers (CRP, TNF-*α*, ESR, and IL-6) when autoantibodies or pathogens are not present [4].

Despite recent scientific achievements, the exact molecular pathophysiology of CRMO is only incompletely understood. Generally, familial (or monogenic) diseases including CRMO as a descriptive symptom can be differentiated from the entity of sporadic CRMO, by other disease features [5].

Differential diagnosis of CRMO is painful bone tumors or lesions such as bacterial osteomyelitis, Ewing sarcoma, leukemia, lymphoma, rhabdomyosarcoma, metastasis of neuroblastoma, eosinophilic granuloma, or Langerhans cell histiocytosis [6–9].

Imaging techniques are essentially important for diagnosing CRMO. Inflammatory bone lesions may be normal in early stages; but in later stages, in plain radiographs, it is detected as radiolucent, sclerotic, or osteolytic lesions. In the early stage of the disease, MRI is highly sensitive [5].

Treatment of patients with CNO/CRMO is based mainly on expert opinion and relatively small case collections. It usually involves nonsteroidal anti-inflammatory drugs (NSAIDs), corticosteroids, disease-modifying antirheumatic drugs (DMARDs, usually methotrexate or sulfasalazine), anti-TNF agents, or bisphosphonates [5].

This study was planned to evaluate the relationship between CRMO and *MEFV* gene mutations as a possible immunopathogenic background in idiopathic forms of CRMO.

## 2. Methods

This study included nine patients diagnosed with idiopathic CRMO at Bu Ali Hospital from 2011 to 2018. Data obtained from their hospital file and after taking confirmed consent form, twelve common *MEFV* gene mutations consisting of E148Q in exon 2, P369S in exon 3, F479L in exon 5, M680I (G/C), M680I (G/A), I692del, M694V, M694I, K695R, V726A, A744S, and R761H in exon 10, were determined to investigate the prevalence of these mutations.

The samples were screened by an RDB assay (FMF StripAssay, Vienna Lab, Vienna, Austria) according to the manufacturer's instructions.

In this study, after excluding primary diseases, we took a thorough history of the patients and performed a complete physical examination, plain radiography, MRI, whole body bone scan with Tc-99 m MDP, and biopsy (in some patients) to diagnose CRMO.

All patients and their first-degree families were screened for the FMF signs and symptoms to exclude any background of Mediterranean fever.

The study was approved by the local ethical committee with the number IR.ARUMS.REC.1396.261, and informed consent was obtained from all the participants.

## 3. Results

Nine patients enrolled in this study, and eight of them were male. The mean age of male patients was 6.9 years and female was 16 years, and the average age of all patients was 9.7 years. The most frequent sites of involvement were tibia and femur, and the least places were zygoma, calcaneus, and radius bones (Table 1).

None of the patients had underlying diseases. Six patients (66.7%) had only one area of involvement. Two patients (22.2%) had two locales, and one patient (11.1%) had three affected regions. Two patients (male) showed positive *MEFV* gene mutations.

The first patient had an E148Q/wt, and the second one showed a K695R/wt as heterozygote mutation (Table 2). There was no significant relationship between the *MEFV* mutations regarding the gender.

Radius and femur were dominant bones involved in patients with positive mutations and patients without mutations, and the most frequent involvement sites were tibia and femur.

There was no significant relationship between the involved bones and MEFV gene mutations.

Regarding the duration of involvement, the average duration in male was 1.3 years and in female was 1.0 and in total was 1.2 years.

Patients with positive mutation had more involved sites and long duration of involvement significantly.

## 4. Discussion

CRMO is an uncommon type of osteomyelitis, presenting in 2–5% of all diagnoses. It is characterized by recurrent periods of focal, chronic, and multiple noninfectious, inflammation of the bone along with exacerbations and remissions.

Microorganisms including atypical mycobacteria, anaerobic streptococci, coagulase-negative staphylococci, *Propionibacterium acnes* have been suggested as the cause, but the etiology of CRMO is yet unknown [10].

It is worth bearing in mind that *P. acnes* is a common organism in the skin and that any biopsy of the bone can also contain some skin. Furthermore, *P. acnes* are commonly detected as a contaminant in microbiological cultures, so its role in CRMO is unclear [11].

Bone pain is usually the initial symptom, but the clinical presentation of CRMO is variable [12].

CRMO can be associated with other inflammatory diseases, such as SAPHO syndrome (synovitis, acne, pustulosis, hyperostosis, and osteitis), psoriasis, IBD (Crohn's disease and ulcerative colitis), peripheral arthritis, sacroiliitis, palmoplantar pustulosis and vasculitis, and granulomatosis with polyangiitis. Majeed syndrome (characterized by CRMO and congenital dyserythropoietic anemia) and Sweet syndrome (acute febrile neutrophilic dermatosis) are other accompanying disorders reported with CRMO [12–16].

Manson et al. [17] suggested diagnostic criteria which are shown in Table 3. The important feature of disease was the plurality of single or multiple lesions [18]. This study included patients with a single lesion.

Autoinflammatory mechanisms are considered as a probable cause of CRMO.

The level of TNF- (tumor necrosis factor-) alpha was reported to increase locally and systemically in patients with active disease [2], suggesting a possible immune-mediated etiology [11].

Instability between proinflammatory and anti-inflammatory cytokines [19], and also increased IL-1*β* secretion by peripheral mononuclear cells of patients with CRMO during active disease [20], is known as an immune-pathogenic process. IL-1 blocking agents and TNF-*α* inhibitors can be used in treatment because IL-1 and TNF-*α* are essential in the pathogenesis of the disease [10].

The distinctive feature of CRMO is aseptic osteitis. However, a negative culture cannot definitively rule out an infective etiology. It can be difficult to diagnose when only one site is involved. In these cases, a biopsy may be carried out to exclude neoplastic and infectious diseases [21].

Biopsy and histological examination can support the diagnosis but are not specific.

CRMO shares many histologic features with acute osteomyelitis such as granulocytic infiltration within affected lesions. The infiltrates mainly consist of lymphocytes, granulocytes, plasma cells, and histiocytes [22, 23].

The prognosis generally is good, and symptom resolution is seen in most patients until adulthood [21, 24]. Some studies have shown that up to 25% of patients have residual disease that leads to skeletal deformity or painless limp [24, 25].

Until recently, the *MEFV* gene had been considered to be responsible only for FMF, but many reports show that the *MEFV* gene is associated with additional clinical presentations within the family of the autoinflammatory diseases such as recurrent monoarthritis, Behcet disease, rheumatoid arthritis, and multiple sclerosis [26–28].

Recent reports of CRMO in the FMF patient [29] and colchicine-responsive CRMO that introduced multifocal osteomyelitis as a variant of familial Mediterranean fever [30] elicited the possible relation between FMF, *MEFV* gene, and CRMO.

In this study, the prevalence of the *MEFV* gene mutations in CRMO patients was about 22%, which is not significantly different from the mutation prevalence in the normal population (25%) of this area (under published data).

In this study, CRMO is significantly higher in males, while in other case series, the ratio of female to male has been higher [10]. There was no significant relationship between age of onset of symptoms and MEFV gene mutations; additionally regarding the sex, age, and area of involvement, there was no any meaning full finding.

These common inflammatory *MEFV* gene mutations seem to have no role in CRMO pathogenesis, and it is unlikely that CRMO is to be a common clinical feature and or the first presentation of FMF patients.

## 5. Conclusion

Although CRMO is an AID, it seems that the *MEFV* gene does not play any role in this inflammation, and it is unlikely that CRMO is presenting feature of FMF disease.

## Figures and Tables

**Table 1:** Locations of involvement.

Percentage	Patients	Locations
11.1	1	Zygoma
22.2	2	Femur
11.1	1	Tibia
11.1	1	Radius
11.1	1	Calcaneus
22.2	2	Two locations
—	1	(i) Tibia and fibula
—	1	(ii) Femur and ischiopubic joint
11.1	1	Three locations
—	1	Humerus, mandible and femur
100	9	Total

**Table 2:** Summary of patient findings.

	Sex	Age/year	Age onset of symptom	Involved sites	Mutations
1	F	17	5	1	—
2	M	4	2	1	—
3	M	13	4	3	—
4	M	10	9	1	—
5	M	11	6	1	—
6	M	10	4	1	K695R/wt
7	M	11	7	2	—
8	M	8	6	2	E148Q/wt
9	M	11	10	1	—

**Table 3:** Proposed criteria for CRMO diagnosis.

Multifocal bone lesions
Remission and exacerbation of signs and symptoms for at least 6 months
Lack of an identifiable cause
Lack of response to antibiotics for at least 1 month
Chronic, nonspecific inflammation consisting of lymphocytes, plasma cells, and histiocytes at histopathologic examination

## Data Availability

The data used to support the findings of this study are included within the article.
